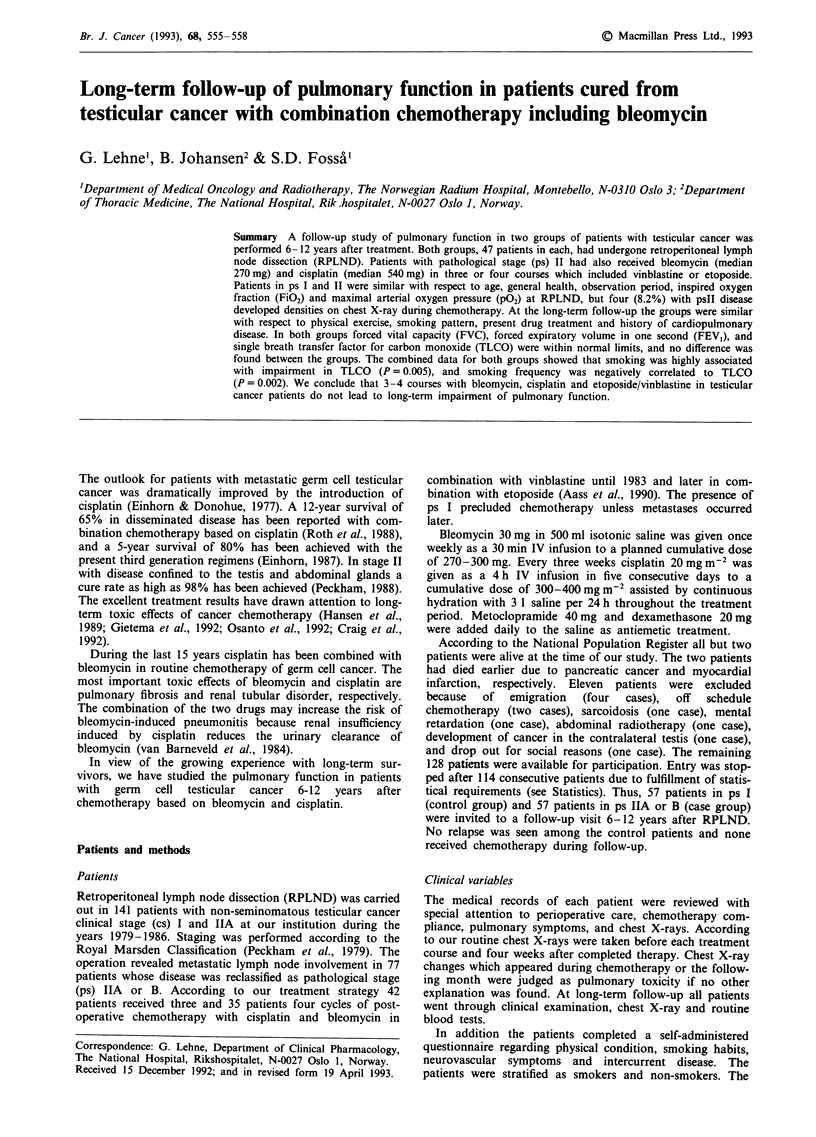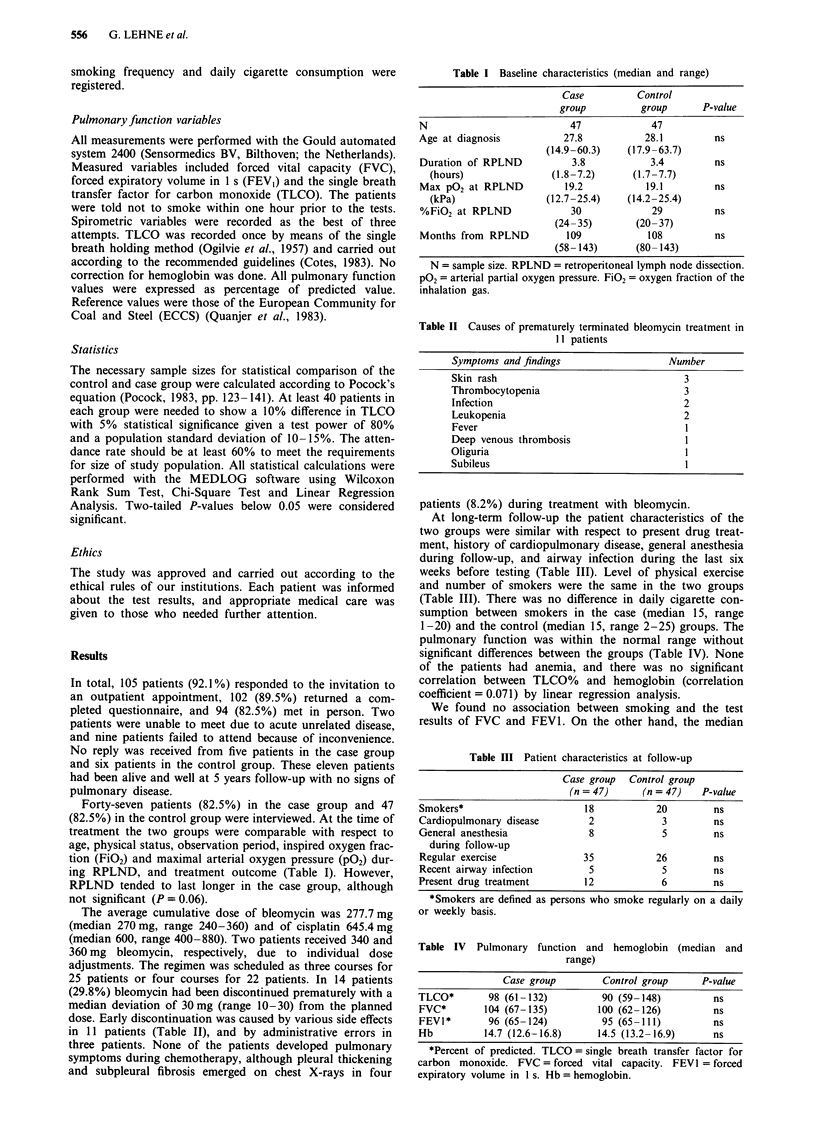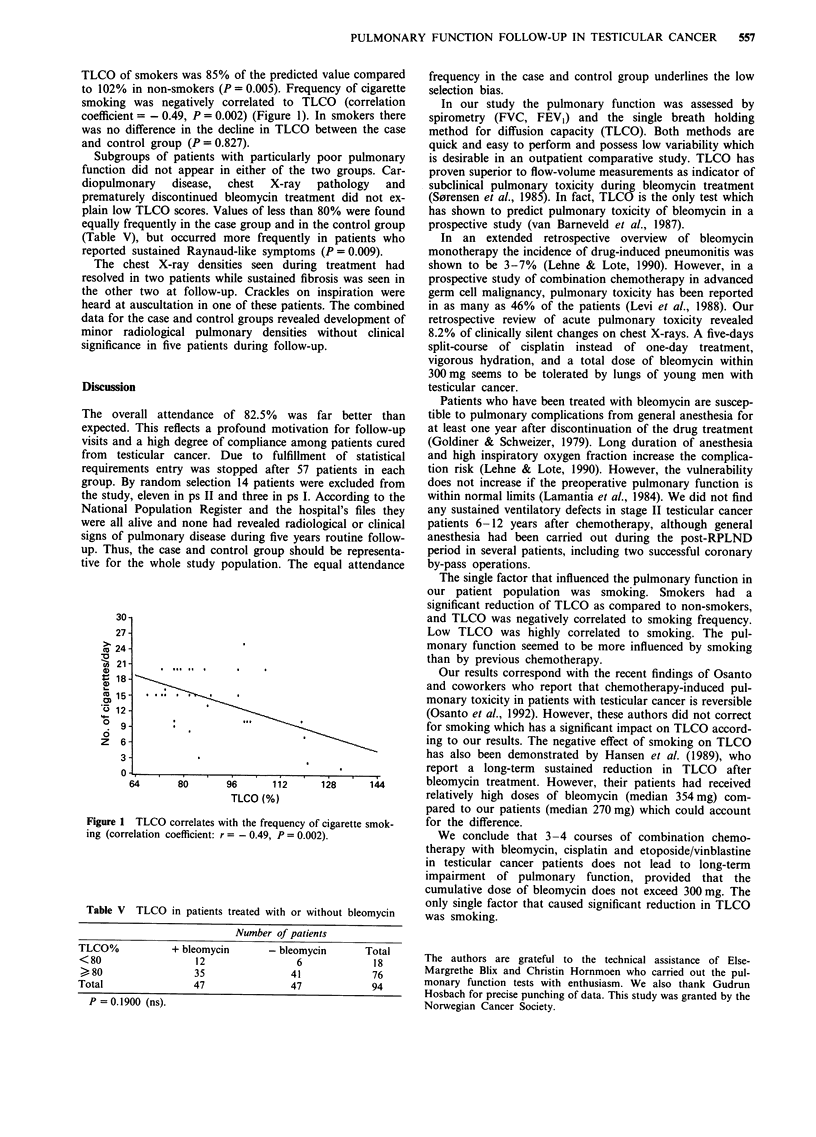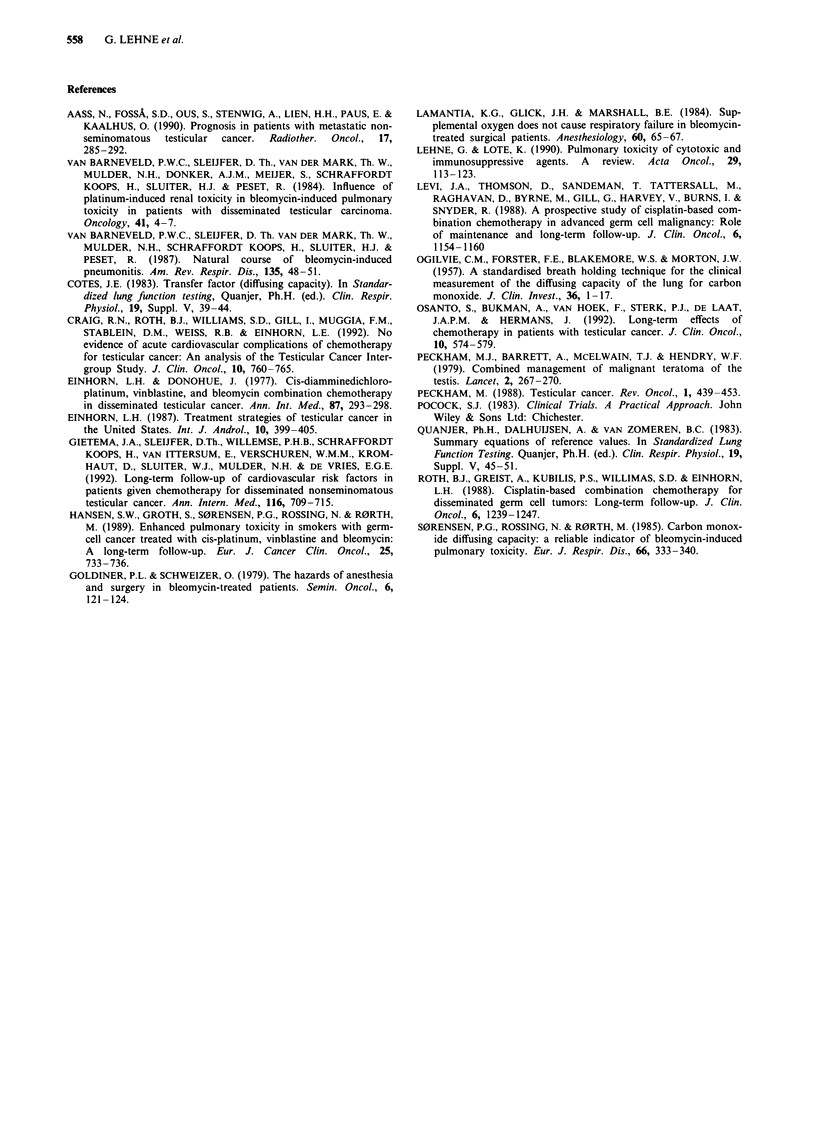# Long-term follow-up of pulmonary function in patients cured from testicular cancer with combination chemotherapy including bleomycin.

**DOI:** 10.1038/bjc.1993.385

**Published:** 1993-09

**Authors:** G. Lehne, B. Johansen, S. D. Fosså

**Affiliations:** Department of Medical Oncology and Radiotherapy, Norwegian Radium Hospital, Montebello, Oslo.

## Abstract

A follow-up study of pulmonary function in two groups of patients with testicular cancer was performed 6-12 years after treatment. Both groups, 47 patients in each, had undergone retroperitoneal lymph node dissection (RPLND). Patients with pathological stage (ps) II had also received bleomycin (median 270 mg) and cisplatin (median 540 mg) in three or four courses which included vinblastine or etoposide. Patients in ps I and II were similar with respect to age, general health, observation period, inspired oxygen fraction (FiO2) and maximal arterial oxygen pressure (pO2) at RPLND, but four (8.2%) with psII disease developed densities on chest X-ray during chemotherapy. At the long-term follow-up the groups were similar with respect to physical exercise, smoking pattern, present drug treatment and history of cardiopulmonary disease. In both groups forced vital capacity (FVC), forced expiratory volume in one second (FEV1), and single breath transfer factor for carbon monoxide (TLCO) were within normal limits, and no difference was found between the groups. The combined data for both groups showed that smoking was highly associated with impairment in TLCO (P = 0.005), and smoking frequency was negatively correlated to TLCO (P = 0.002). We conclude that 3-4 courses with bleomycin, cisplatin and etoposide/vinblastine in testicular cancer patients do not lead to long-term impairment of pulmonary function.


Br. J. Cancer (1993), 68, 555-558 © Macmillan Press Ltd., 1993

### Long-term follow-up of pulmonary function in patients cured from testicular cancer with combination chemotherapy including bleomycin

G. Lehne[1], B. Johansen[2] & S.D. Fosså[1]

[1]*Department of Medical Oncology and Radiotherapy, The Norwegian Radium Hospital, Montebello, N-0310 Oslo 3;* [2]*Department of Thoracic Medicine, The National Hospital, Rik.hospitalet, N-0027 Oslo 1, Norway.*

**Summary** A follow-up study of pulmonary function in two groups of patients with testicular cancer was performed 6-12 years after treatment. Both groups, 47 patients in each, had undergone retroperitoneal lymph node dissection (RPLND). Patients with pathological stage (ps) II had also received bleomycin (median 270 mg) and cisplatin (median 540 mg) in three or four courses which included vinblastine or etoposide. Patients in ps I and II were similar with respect to age, general health, observation period, inspired oxygen fraction ($FiO_2$) and maximal arterial oxygen pressure ($pO_2$) at RPLND, but four (8.2%) with psII disease developed densities on chest X-ray during chemotherapy. At the long-term follow-up the groups were similar with respect to physical exercise, smoking pattern, present drug treatment and history of cardiopulmonary disease. In both groups forced vital capacity (FVC), forced expiratory volume in one second ($FEV_1$), and single breath transfer factor for carbon monoxide (TLCO) were within normal limits, and no difference was found between the groups. The combined data for both groups showed that smoking was highly associated with impairment in TLCO ($P = 0.005$), and smoking frequency was negatively correlated to TLCO ($P = 0.002$). We conclude that 3-4 courses with bleomycin, cisplatin and etoposide/vinblastine in testicular cancer patients do not lead to long-term impairment of pulmonary function.

The outlook for patients with metastatic germ cell testicular cancer was dramatically improved by the introduction of cisplatin (Einhorn & Donohue, 1977). A 12-year survival of 65% in disseminated disease has been reported with combination chemotherapy based on cisplatin (Roth *et al.*, 1988), and a 5-year survival of 80% has been achieved with the present third generation regimens (Einhorn, 1987). In stage II with disease confined to the testis and abdominal glands a cure rate as high as 98% has been achieved (Peckham, 1988). The excellent treatment results have drawn attention to long-term toxic effects of cancer chemotherapy (Hansen *et al.*, 1989; Gietema *et al.*, 1992; Osanto *et al.*, 1992; Craig *et al.*, 1992).

During the last 15 years cisplatin has been combined with bleomycin in routine chemotherapy of germ cell cancer. The most important toxic effects of bleomycin and cisplatin are pulmonary fibrosis and renal tubular disorder, respectively. The combination of the two drugs may increase the risk of bleomycin-induced pneumonitis because renal insufficiency induced by cisplatin reduces the urinary clearance of bleomycin (van Barneveld *et al.*, 1984).

In view of the growing experience with long-term survivors, we have studied the pulmonary function in patients with germ cell testicular cancer 6-12 years after chemotherapy based on bleomycin and cisplatin.

### Patients and methods

### *Patients*

Retroperitoneal lymph node dissection (RPLND) was carried out in 141 patients with non-seminomatous testicular cancer clinical stage (cs) I and IIA at our institution during the years 1979-1986. Staging was performed according to the Royal Marsden Classification (Peckham *et al.*, 1979). The operation revealed metastatic lymph node involvement in 77 patients whose disease was reclassified as pathological stage (ps) IIA or B. According to our treatment strategy 42 patients received three and 35 patients four cycles of post-operative chemotherapy with cisplatin and bleomycin in combination with vinblastine until 1983 and later in combination with etoposide (Aass *et al.*, 1990). The presence of ps I precluded chemotherapy unless metastases occurred later.

Bleomycin 30 mg in 500 ml isotonic saline was given once weekly as a 30 min IV infusion to a planned cumulative dose of 270-300 mg. Every three weeks cisplatin 20 mg $m^{-2}$ was given as a 4 h IV infusion in five consecutive days to a cumulative dose of 300-400 mg $m^{-2}$ assisted by continuous hydration with 3 l saline per 24 h throughout the treatment period. Metoclopramide 40 mg and dexamethasone 20 mg were added daily to the saline as antiemetic treatment.

According to the National Population Register all but two patients were alive at the time of our study. The two patients had died earlier due to pancreatic cancer and myocardial infarction, respectively. Eleven patients were excluded because of emigration (four cases), off schedule chemotherapy (two cases), sarcoidosis (one case), mental retardation (one case), abdominal radiotherapy (one case), development of cancer in the contralateral testis (one case), and drop out for social reasons (one case). The remaining 128 patients were available for participation. Entry was stopped after 114 consecutive patients due to fulfillment of statistical requirements (see Statistics). Thus, 57 patients in ps I (control group) and 57 patients in ps IIA or B (case group) were invited to a follow-up visit 6-12 years after RPLND. No relapse was seen among the control patients and none received chemotherapy during follow-up.

### *Clinical variables*

The medical records of each patient were reviewed with special attention to perioperative care, chemotherapy compliance, pulmonary symptoms, and chest X-rays. According to our routine chest X-rays were taken before each treatment course and four weeks after completed therapy. Chest X-ray changes which appeared during chemotherapy or the following month were judged as pulmonary toxicity if no other explanation was found. At long-term follow-up all patients went through clinical examination, chest X-ray and routine blood tests.

In addition the patients completed a self-administered questionnaire regarding physical condition, smoking habits, neurovascular symptoms and intercurrent disease. The patients were stratified as smokers and non-smokers. The

Correspondence: G. Lehne, Department of Clinical Pharmacology, The National Hospital, Rikshospitalet, N-0027 Oslo 1, Norway.
Received 15 December 1992; and in revised form 19 April 1993.

smoking frequency and daily cigarette consumption were registered.

*Pulmonary function variables*

All measurements were performed with the Gould automated system 2400 (Sensormedics BV, Bilthoven; the Netherlands). Measured variables included forced vital capacity (FVC), forced expiratory volume in 1 s ($FEV_1$) and the single breath transfer factor for carbon monoxide (TLCO). The patients were told not to smoke within one hour prior to the tests. Spirometric variables were recorded as the best of three attempts. TLCO was recorded once by means of the single breath holding method (Ogilvie *et al.*, 1957) and carried out according to the recommended guidelines (Cotes, 1983). No correction for hemoglobin was done. All pulmonary function values were expressed as percentage of predicted value. Reference values were those of the European Community for Coal and Steel (ECCS) (Quanjer *et al.*, 1983).

*Statistics*

The necessary sample sizes for statistical comparison of the control and case group were calculated according to Pocock's equation (Pocock, 1983, pp. 123–141). At least 40 patients in each group were needed to show a 10% difference in TLCO with 5% statistical significance given a test power of 80% and a population standard deviation of 10–15%. The attendance rate should be at least 60% to meet the requirements for size of study population. All statistical calculations were performed with the MEDLOG software using Wilcoxon Rank Sum Test, Chi-Square Test and Linear Regression Analysis. Two-tailed $P$-values below 0.05 were considered significant.

*Ethics*

The study was approved and carried out according to the ethical rules of our institutions. Each patient was informed about the test results, and appropriate medical care was given to those who needed further attention.

### Results

In total, 105 patients (92.1%) responded to the invitation to an outpatient appointment, 102 (89.5%) returned a completed questionnaire, and 94 (82.5%) met in person. Two patients were unable to meet due to acute unrelated disease, and nine patients failed to attend because of inconvenience. No reply was received from five patients in the case group and six patients in the control group. These eleven patients had been alive and well at 5 years follow-up with no signs of pulmonary disease.

Forty-seven patients (82.5%) in the case group and 47 (82.5%) in the control group were interviewed. At the time of treatment the two groups were comparable with respect to age, physical status, observation period, inspired oxygen fraction ($FiO_2$) and maximal arterial oxygen pressure ($pO_2$) during RPLND, and treatment outcome (Table I). However, RPLND tended to last longer in the case group, although not significant ($P = 0.06$).

The average cumulative dose of bleomycin was 277.7 mg (median 270 mg, range 240–360) and of cisplatin 645.4 mg (median 600, range 400–880). Two patients received 340 and 360 mg bleomycin, respectively, due to individual dose adjustments. The regimen was scheduled as three courses for 25 patients or four courses for 22 patients. In 14 patients (29.8%) bleomycin had been discontinued prematurely with a median deviation of 30 mg (range 10–30) from the planned dose. Early discontinuation was caused by various side effects in 11 patients (Table II), and by administrative errors in three patients. None of the patients developed pulmonary symptoms during chemotherapy, although pleural thickening and subpleural fibrosis emerged on chest X-rays in four patients (8.2%) during treatment with bleomycin.

**Table I** Baseline characteristics (median and range)

| | *Case group* | *Control group* | *P-value* |
|---|---|---|---|
| N | 47 | 47 | |
| Age at diagnosis | 27.8 (14.9–60.3) | 28.1 (17.9–63.7) | ns |
| Duration of RPLND (hours) | 3.8 (1.8–7.2) | 3.4 (1.7–7.7) | ns |
| Max $pO_2$ at RPLND (kPa) | 19.2 (12.7–25.4) | 19.1 (14.2–25.4) | ns |
| %$FiO_2$ at RPLND | 30 (24–35) | 29 (20–37) | ns |
| Months from RPLND | 109 (58–143) | 108 (80–143) | ns |

N = sample size. RPLND = retroperitoneal lymph node dissection. $pO_2$ = arterial partial oxygen pressure. $FiO_2$ = oxygen fraction of the inhalation gas.

**Table II** Causes of prematurely terminated bleomycin treatment in 11 patients

| *Symptoms and findings* | *Number* |
|---|---|
| Skin rash | 3 |
| Thrombocytopenia | 3 |
| Infection | 2 |
| Leukopenia | 2 |
| Fever | 1 |
| Deep venous thrombosis | 1 |
| Oliguria | 1 |
| Subileus | 1 |

At long-term follow-up the patient characteristics of the two groups were similar with respect to present drug treatment, history of cardiopulmonary disease, general anesthesia during follow-up, and airway infection during the last six weeks before testing (Table III). Level of physical exercise and number of smokers were the same in the two groups (Table III). There was no difference in daily cigarette consumption between smokers in the case (median 15, range 1–20) and the control (median 15, range 2–25) groups. The pulmonary function was within the normal range without significant differences between the groups (Table IV). None of the patients had anemia, and there was no significant correlation between TLCO% and hemoglobin (correlation coefficient = 0.071) by linear regression analysis.

We found no association between smoking and the test results of FVC and FEV1. On the other hand, the median

**Table III** Patient characteristics at follow-up

| | *Case group (n = 47)* | *Control group (n = 47)* | *P-value* |
|---|---|---|---|
| Smokers* | 18 | 20 | ns |
| Cardiopulmonary disease | 2 | 3 | ns |
| General anesthesia during follow-up | 8 | 5 | ns |
| Regular exercise | 35 | 26 | ns |
| Recent airway infection | 5 | 5 | ns |
| Present drug treatment | 12 | 6 | ns |

*Smokers are defined as persons who smoke regularly on a daily or weekly basis.

**Table IV** Pulmonary function and hemoglobin (median and range)

| | *Case group* | *Control group* | *P-value* |
|---|---|---|---|
| TLCO* | 98 (61–132) | 90 (59–148) | ns |
| FVC* | 104 (67–135) | 100 (62–126) | ns |
| FEV1* | 96 (65–124) | 95 (65–111) | ns |
| Hb | 14.7 (12.6–16.8) | 14.5 (13.2–16.9) | ns |

*Percent of predicted. TLCO = single breath transfer factor for carbon monoxide. FVC = forced vital capacity. FEV1 = forced expiratory volume in 1 s. Hb = hemoglobin.

TLCO of smokers was 85% of the predicted value compared to 102% in non-smokers ($P = 0.005$). Frequency of cigarette smoking was negatively correlated to TLCO (correlation coefficient = – 0.49, $P = 0.002$) (Figure 1). In smokers there was no difference in the decline in TLCO between the case and control group ($P = 0.827$).

Subgroups of patients with particularly poor pulmonary function did not appear in either of the two groups. Cardiopulmonary disease, chest X-ray pathology and prematurely discontinued bleomycin treatment did not explain low TLCO scores. Values of less than 80% were found equally frequently in the case group and in the control group (Table V), but occurred more frequently in patients who reported sustained Raynaud-like symptoms ($P = 0.009$).

The chest X-ray densities seen during treatment had resolved in two patients while sustained fibrosis was seen in the other two at follow-up. Crackles on inspiration were heard at auscultation in one of these patients. The combined data for the case and control groups revealed development of minor radiological pulmonary densities without clinical significance in five patients during follow-up.

### Discussion

The overall attendance of 82.5% was far better than expected. This reflects a profound motivation for follow-up visits and a high degree of compliance among patients cured from testicular cancer. Due to fulfillment of statistical requirements entry was stopped after 57 patients in each group. By random selection 14 patients were excluded from the study, eleven in ps II and three in ps I. According to the National Population Register and the hospital's files they were all alive and none had revealed radiological or clinical signs of pulmonary disease during five years routine follow-up. Thus, the case and control group should be representative for the whole study population. The equal attendance frequency in the case and control group underlines the low selection bias.

**Figure 1** TLCO correlates with the frequency of cigarette smoking (correlation coefficient: $r = -0.49$, $P = 0.002$).

**Table V** TLCO in patients treated with or without bleomycin

| | Number of patients | | |
|---|---|---|---|
| TLCO% | + bleomycin | – bleomycin | Total |
| <80 | 12 | 6 | 18 |
| ⩾80 | 35 | 41 | 76 |
| Total | 47 | 47 | 94 |

$P = 0.1900$ (ns).

In our study the pulmonary function was assessed by spirometry (FVC, $FEV_1$) and the single breath holding method for diffusion capacity (TLCO). Both methods are quick and easy to perform and possess low variability which is desirable in an outpatient comparative study. TLCO has proven superior to flow-volume measurements as indicator of subclinical pulmonary toxicity during bleomycin treatment (Sørensen *et al.*, 1985). In fact, TLCO is the only test which has shown to predict pulmonary toxicity of bleomycin in a prospective study (van Barneveld *et al.*, 1987).

In an extended retrospective overview of bleomycin monotherapy the incidence of drug-induced pneumonitis was shown to be 3–7% (Lehne & Lote, 1990). However, in a prospective study of combination chemotherapy in advanced germ cell malignancy, pulmonary toxicity has been reported in as many as 46% of the patients (Levi *et al.*, 1988). Our retrospective review of acute pulmonary toxicity revealed 8.2% of clinically silent changes on chest X-rays. A five-days split-course of cisplatin instead of one-day treatment, vigorous hydration, and a total dose of bleomycin within 300 mg seems to be tolerated by lungs of young men with testicular cancer.

Patients who have been treated with bleomycin are susceptible to pulmonary complications from general anesthesia for at least one year after discontinuation of the drug treatment (Goldiner & Schweizer, 1979). Long duration of anesthesia and high inspiratory oxygen fraction increase the complication risk (Lehne & Lote, 1990). However, the vulnerability does not increase if the preoperative pulmonary function is within normal limits (Lamantia *et al.*, 1984). We did not find any sustained ventilatory defects in stage II testicular cancer patients 6–12 years after chemotherapy, although general anesthesia had been carried out during the post-RPLND period in several patients, including two successful coronary by-pass operations.

The single factor that influenced the pulmonary function in our patient population was smoking. Smokers had a significant reduction of TLCO as compared to non-smokers, and TLCO was negatively correlated to smoking frequency. Low TLCO was highly correlated to smoking. The pulmonary function seemed to be more influenced by smoking than by previous chemotherapy.

Our results correspond with the recent findings of Osanto and coworkers who report that chemotherapy-induced pulmonary toxicity in patients with testicular cancer is reversible (Osanto *et al.*, 1992). However, these authors did not correct for smoking which has a significant impact on TLCO according to our results. The negative effect of smoking on TLCO has also been demonstrated by Hansen *et al.* (1989), who report a long-term sustained reduction in TLCO after bleomycin treatment. However, their patients had received relatively high doses of bleomycin (median 354 mg) compared to our patients (median 270 mg) which could account for the difference.

We conclude that 3–4 courses of combination chemotherapy with bleomycin, cisplatin and etoposide/vinblastine in testicular cancer patients does not lead to long-term impairment of pulmonary function, provided that the cumulative dose of bleomycin does not exceed 300 mg. The only single factor that caused significant reduction in TLCO was smoking.

The authors are grateful to the technical assistance of Else-Margrethe Blix and Christin Hornmoen who carried out the pulmonary function tests with enthusiasm. We also thank Gudrun Hosbach for precise punching of data. This study was granted by the Norwegian Cancer Society.

### References

AASS, N., FOSSÅ, S.D., OUS, S., STENWIG, A., LIEN, H.H., PAUS, E. & KAALHUS, O. (1990). Prognosis in patients with metastatic non-seminomatous testicular cancer. *Radiother. Oncol.*, **17**, 285–292.

VAN BARNEVELD, P.W.C., SLEIJFER, D. Th., VAN DER MARK, Th. W., MULDER, N.H., DONKER, A.J.M., MEIJER, S., SCHRAFFORDT KOOPS, H., SLUITER, H.J. & PESET, R. (1984). Influence of platinum-induced renal toxicity in bleomycin-induced pulmonary toxicity in patients with disseminated testicular carcinoma. *Oncology*, **41**, 4–7.

VAN BARNEVELD, P.W.C., SLEIJFER, D. Th. VAN DER MARK, Th. W., MULDER, N.H., SCHRAFFORDT KOOPS, H., SLUITER, H.J. & PESET, R. (1987). Natural course of bleomycin-induced pneumonitis. *Am. Rev. Respir. Dis.*, **135**, 48–51.

COTES, J.E. (1983). Transfer factor (diffusing capacity). In *Standardized lung function testing*, Quanjer, Ph.H. (ed.). *Clin. Respir. Physiol.*, **19**, Suppl. V, 39–44.

CRAIG, R.N., ROTH, B.J., WILLIAMS, S.D., GILL, I., MUGGIA, F.M., STABLEIN, D.M., WEISS, R.B. & EINHORN, L.E. (1992). No evidence of acute cardiovascular complications of chemotherapy for testicular cancer: An analysis of the Testicular Cancer Intergroup Study. *J. Clin. Oncol.*, **10**, 760–765.

EINHORN, L.H. & DONOHUE, J. (1977). Cis-diamminedichloroplatinum, vinblastine, and bleomycin combination chemotherapy in disseminated testicular cancer. *Ann. Int. Med.*, **87**, 293–298.

EINHORN, L.H. (1987). Treatment strategies of testicular cancer in the United States. *Int. J. Androl.*, **10**, 399–405.

GIETEMA, J.A., SLEIJFER, D.Th., WILLEMSE, P.H.B., SCHRAFFORDT KOOPS, H., VAN ITTERSUM, E., VERSCHUREN, W.M.M., KROMHAUT, D., SLUITER, W.J., MULDER, N.H. & DE VRIES, E.G.E. (1992). Long-term follow-up of cardiovascular risk factors in patients given chemotherapy for disseminated nonseminomatous testicular cancer. *Ann. Intern. Med.*, **116**, 709–715.

HANSEN, S.W., GROTH, S., SØRENSEN, P.G., ROSSING, N. & RØRTH, M. (1989). Enhanced pulmonary toxicity in smokers with germ-cell cancer treated with cis-platinum, vinblastine and bleomycin: A long-term follow-up. *Eur. J. Cancer Clin. Oncol.*, **25**, 733–736.

GOLDINER, P.L. & SCHWEIZER, O. (1979). The hazards of anesthesia and surgery in bleomycin-treated patients. *Semin. Oncol.*, **6**, 121–124.

LAMANTIA, K.G., GLICK, J.H. & MARSHALL, B.E. (1984). Supplemental oxygen does not cause respiratory failure in bleomycin-treated surgical patients. *Anesthesiology*, **60**, 65–67.

LEHNE, G. & LOTE, K. (1990). Pulmonary toxicity of cytotoxic and immunosuppressive agents. A review. *Acta Oncol.*, **29**, 113–123.

LEVI, J.A., THOMSON, D., SANDEMAN, T. TATTERSALL, M., RAGHAVAN, D., BYRNE, M., GILL, G., HARVEY, V., BURNS, I. & SNYDER, R. (1988). A prospective study of cisplatin-based combination chemotherapy in advanced germ cell malignancy: Role of maintenance and long-term follow-up. *J. Clin. Oncol.*, **6**, 1154–1160

OGILVIE, C.M., FORSTER, F.E., BLAKEMORE, W.S. & MORTON, J.W. (1957). A standardised breath holding technique for the clinical measurement of the diffusing capacity of the lung for carbon monoxide. *J. Clin. Invest.*, **36**, 1–17.

OSANTO, S., BUKMAN, A., VAN HOEK, F., STERK, P.J., DE LAAT, J.A.P.M. & HERMANS, J. (1992). Long-term effects of chemotherapy in patients with testicular cancer. *J. Clin. Oncol.*, **10**, 574–579.

PECKHAM, M.J., BARRETT, A., MCELWAIN, T.J. & HENDRY, W.F. (1979). Combined management of malignant teratoma of the testis. *Lancet*, **2**, 267–270.

PECKHAM, M. (1988). Testicular cancer. *Rev. Oncol.*, **1**, 439–453.

POCOCK, S.J. (1983). *Clinical Trials. A Practical Approach*. John Wiley & Sons Ltd: Chichester.

QUANJER, Ph.H., DALHUIJSEN, A. & VAN ZOMEREN, B.C. (1983). Summary equations of reference values. In *Standardized Lung Function Testing*. Quanjer, Ph.H. (ed.). *Clin. Respir. Physiol.*, **19**, Suppl. V, 45–51.

ROTH, B.J., GREIST, A., KUBILIS, P.S., WILLIMAS, S.D. & EINHORN, L.H. (1988). Cisplatin-based combination chemotherapy for disseminated germ cell tumors: Long-term follow-up. *J. Clin. Oncol.*, **6**, 1239–1247.

SØRENSEN, P.G., ROSSING, N. & RØRTH, M. (1985). Carbon monoxide diffusing capacity: a reliable indicator of bleomycin-induced pulmonary toxicity. *Eur. J. Respir. Dis.*, **66**, 333–340.